# Effect of *Scoparia dulcis* Extract on Lipid Oxidation in Fish Feed, Growth Performance, and Hypoxia Tolerance in Juvenile Jian Carp (*Cyprinus carpio* var. Jian)

**DOI:** 10.1155/2024/7432096

**Published:** 2024-05-17

**Authors:** Gangfu Chen, Jing Xu, Fengyi Li, Mingquan He, Xiaolu Yu, Wenhao Du, Yuxue Ye, Ling Liao, Min Wu, Huatao Li

**Affiliations:** Key Laboratory of Sichuan Province for Conservation and Utilization of Fishes Resources in the Upper Reaches of the Yangtze River, College of Life Sciences, Neijiang Normal University, Neijiang, Sichuan 641100, China

## Abstract

Lipid oxidation and hypoxia can lead to oxidative damage in aquatic animals. This study explored the effects of *Scoparia dulcis* extracts (SDE) on lipid oxidation, fish growth performance, digestive ability, antioxidant capacity, and hypoxia tolerance ability. The results showed that SDE decreased malonaldehyde (MDA), conjugated diene (CD), and peroxide value (PO) in the linoleic acid and linolenic acid as well as in fish feed. Broken-line analysis revealed that the optimal acetone extract of *S. dulcis* (AE) supplements was 4.02, 4.01, and 4.01 g kg^−1^ determined from PO, CD, and MDA, respectively. Dietary AE supplementation increased feed intake and specific growth rate and activities of amylase, trypsin, and lipase as well as alkaline phosphatase in fish hepatopancreas and gut. Polynomial regression analysis showed that optimal dietary AE supplement was 3.61 g kg^−1^ diet determined from weight gain. Furthermore, dietary AE supplementation decreased MDA content and increased glutathione content and the activities of glutathione peroxidase, catalase, superoxide dismutase, and glutathione reductase in fish digestive organs, gills, erythrocytes, and muscle. Dietary AE supplementation increased durative time (DT) and oxygen consumption rate (OCR) under hypoxia condition. Based on polynomial regression analysis, optimal dietary AE supplements were 4.73 and 4.60 g kg^−1^ diet determined from DT and OCR for hypoxia tolerance in fish, respectively. According to our current research, SDE's antioxidant capacity may be attribute to their phenolic chemicals.

## 1. Introduction

Fish feed has a high fat content and is abundant in fatty acids [1]. Unsaturated fatty acids in fish are readily oxidized to yield oxidation products such as peroxide (PO), malondialdehyde (MDA), and conjugated diene (CD) when subjected to the reactive oxygen species (ROS) or an excess of redox metal ions [2, 3]. The high concentration of polyunsaturated fatty acids (PUFAs) in fish flesh makes it susceptible to lipid oxidation [4]. Blood is not always eliminated from fish during processing, which leaves fish flesh with a high concentration of hemoglobin (Hb) [5]. The auto-oxidation of Hb can produce ROS, causing heme iron to be released from erythrocytes and lipids in fish flesh to oxidize [6]. In addition, the health of animals and growth are negatively impacted by dietary oxidized lipid [7]. Lipid oxidation can damage fish flesh quality by reducing shelf life, producing toxic metabolites, breaking down nutritious components, and affecting color, fragrance, and flavor [8].

In the food, cosmetic, and pharmaceutical industries, artificial antioxidants are widely employed to prevent lipid oxidation [8]. Our team recently in fish feed discovered that butylated hydroxytoluene and ethoxyquin depressed lipid oxidation [9]. Nevertheless, these artificial antioxidants have the potential to accumulate in animal tissues and are hazardous and carcinogenic to animals [10], which can then be consumed by people [8]. Considering the negative consequences on both people and animals, the use of these artificial antioxidants in the food sector should be strictly monitored [11]. Consequently, natural substances show promise as an alternative to artificial antioxidants [12].

The dissolved oxygen (DO) level in water is directly related to fish growth performance [13]. In fish, dissolved oxygen can be transported to different tissues for consumption, and it is a major source of limitation in fish production since they have aerobic metabolism that requires adequate DO [14]. Hypoxia, or oxygen deficiency, is a normal event resulting from daily fluctuations in oxygen content in water [15], leading the fish to experience conditions of hypoxia/reoxygenation frequently. Fish growth performance is linked to their ability to digest and absorb nutrients, as well as their respiratory function [13, 16]. Fish rely heavily on the hepatopancreas and gut for digestion and absorption [17]. The fish gill is the primary organ responsible for respiratory function and maintaining acid–base balance [18]. Fish erythrocytes, carrying hemoglobin (Hb), are essential for the supply of oxygen to all tissues and organs [19]. Nevertheless, hyperoxia elevated ROS production, resulting in oxidative stress in the gills and liver with the histopathological changes in mirror carp (*Cyprinus carpio* L.) [13]. The fish antioxidant defense system, which includes nonenzymatic and enzymatic antioxidants, is critical for combating oxidative stress and protecting against damage [20]. On the one hand, hypoxia decreased fish growth performance [13, 21]; on the other hand, it increased the levels of antioxidant defenses in goldfish (*Carassius auratus*) [22]. Therefore, there is a need to expand our understanding of approaches to mitigate the negative effects induced by hypoxia.

*Scoparia dulcis* L. (*S. dulcis*) is a traditional Chinese medicinal plant with bioactive compounds such as tannins, phenolics, flavonoids, and diterpenoids [23]. Alloxan-induced body weight loss in rats was mitigated when *S. dulcis* extract (SDE) was added to their diet [24]. Our lab has shown that adding SDE to the diet can mitigate the negative impact of density stress on crucian carp (*C. auratus*) growth [16]. Moreover, supplementation with SDE decreased histological abnormalities in the pancreas of MLD-STZ-induced hyperglycemia mice [25]. In mice, the addition of SDE to the diet alleviated paw edema caused by *λ*-carrageenan, indicated by the increase of glutathione peroxidase (GPx) and superoxide dismutase (SOD) activities in the liver and decreased MDA levels [26]. Our recent study observed that adding SDE to the diet relieved the detrimental effects on fish digestive and absorptive abilities, induced by density and copper (Cu) stress, and on antioxidant status which induced by trichlorfon in fish muscles [16].

This investigation was intended to investigate the idea that dietary SDE improves the antioxidant capacity that can relieve the detrimental effects of hypoxia and lipid oxidation. Thus, the current investigation explored how SDE affected lipid oxidation in aquatic feed and fish growth performance and the potential protective effects against hyperoxia. These findings may provide a foundation for employing SDE as a natural antioxidant component for aquatic animals.

## 2. Materials and Methods

### 2.1. Chemical Reagent

Chengdu Kelong Chemical Reagent Factory (Chengdu, China) supplied Tween 20, ethyl acetate, petroleum ether, and acetone, and Shanghai Biochemical Reagent Co., Ltd. (Shanghai, China) provided the linoleic acid (≥97%) and linolenic acid (≥95%). The remaining chemicals employed in this investigation were all of analytical reagent quality.

### 2.2. S. dulcis Extract (SDE) Preparation and Composition Analyses

**Figure 1:**
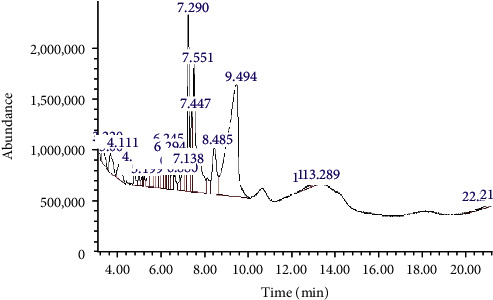
The gas chromatogram of acetone extract of *S. dulcis*. This experiment was repeated three times with similar results achieved.

The SDE preparation processes were carried out in accordance with our group's earlier description [16]. SDE composition analyses were performed according to our previous published method [17]. Results were showed in Figure 1 and Table 1.

### 2.3. Measurement of Phenolic Content

The phenolic concentration, total antioxidant capacity (T-AOC), and metal chelating ability (MCA) of SDE were measured using the techniques of Panyatip et al. [27], Cao et al. [28], and Zhao et al. [29], in that order.

### 2.4. Lipid Oxidation Assessment in Unsaturated Fatty Acids

The effects of SDE on lipid oxidation were measured using the techniques of Yuan et al. [30] with slight modification. Linoleic and linolenic acid (0.1 mL), 9.9 mL of cold phosphate buffer (pH 7.0, 0.02 M), and 50 *μ*L Tween 20 were added to form the emulsion. It was then homogenized on ice using a homogenizer (FJ200-SH, Shanghai, China) for 2 × 10 s at a speed of 21,000x *g*. After dissolving the SDE in the emulsion, the final concentrations were 1.0 mg mL^−1^ and 0 (control). The levels of MDA, CD, and PO were assessed following an 8-day incubation period at 45°C, in accordance with Maqsood and Benjakul's [31] protocol.

### 2.5. Lipid Oxidation Assessment in Fish Feed

The processes of diet preparation were carried out in accordance with our group's earlier description [16]. Seven experimental feeds were formulated with AE from 0.0 to 6.0 g kg^−1^ as described by Chen et al. [32]. The feed was treated similar to the linoleic acid and linolenic acid emulsion as described above.

### 2.6. Feeding Experiment

Jian carps were procured from a local fishery (Neijiang, China). Fish were kept under a natural light condition in laboratory [16]. Juvenile Jian carp (4.3 ± 0.2 g) were stochastically allocated into seven treatments. Each treatment had four duplicate tanks with 20 fish each. For 60 days, AE was added to experimental meals at doses ranging from 0.0 to 6.0 g kg^−1^. Survival rate, weight gain (WG), feed intake (FI), specific growth rate (SGR), and feed efficiency (FE) were calculated according to our previous study [32]. Sample collection protocol was followed as it was done in our previous study [17]. Five fish from each aquarium were anesthetized using benzocaine (50 mg L^−1^), and the hepatopancreas, gut, muscle, and gills were obtained and frozen at liquid nitrogen for further assessment.

### 2.7. Hypoxia Experiment

After the feeding experiment, five fish from each aquarium were used for hypoxia assays according to our previous study [33]. The oxygen consumption rate (OCR) and durative time (DT) were determined.

### 2.8. Parameter Measurement

Activities of amylase, lipase, trypsin, glutamate–oxaloacetate transaminase (GOT), lactate dehydrogenase (LDH), glutamate–pyruvate transaminase (GPT), and alkaline phosphatase (AKP) were assayed. Content of protein carbonyl (PC), glutathione (GSH), and MDA; activities of catalase (CAT), glutathione-*S*-transferase (GST), glutathione peroxidase (GPx), superoxide dismutase (SOD), and glutathione reductase (GR); and capacity of antihydroxyl radical (AHR) and antisuperoxide anion (ASA) were measured. Caudal punctures were used to collect blood into a heparinized syringe from 15 fish of each group. And the blood underwent 3 min of centrifugation treatment at 1,000x *g* at 4°C within 1 hr. The isolation of plasma from erythrocytes was to measure related parameters according to Li et al. [34]. The hematocrit (Hct), red blood corpuscle count (RBC), hemoglobin concentration (HbC), and plasma ammonia content (PAC) were quantified. The mean corpuscular volume (MCV), mean corpuscular hemoglobin concentration (MCHC), and mean corpuscular hemoglobin content (MCH) were computed according to our previous study [33]. In erythrocytes, activities of LDH, GOT, GPT, SOD, and CAT and content of superoxide anion (O_2_^•−^), MDA, GSH, met-hemoglobin (Met-Hb), and hydrogen peroxide (H_2_O_2_) were measured.

### 2.9. Biochemical Analysis

Activities of lipase, AKP, amylase, trypsin, GOT, and GPT were carried out in accordance with the group's earlier description [32]. The capacity of ASA and AHR and the levels of PC, PAC, GSH, and MDA, as well as activities of SOD, CAT, GPx, LDH, and GR, were measured in accordance with Chen et al. [17]. The levels of H_2_O_2_, O_2_^•−^, and Met-Hb were assayed using those described by Li et al. [35]. The activities of GST were measured according to Jiang et al. [4].

### 2.10. Data Analysis

The mean ± standard deviation (SD) is used to express the data. Using SPSS version 15.0, a one-way ANOVA was performed on the data. The statistical significance was compared by utilizing Duncan's multiple range test.

## 3. Results

### 3.1. Relationship between Phenolic Concentration in SDE and Antioxidant Capacity

**Figure 2:**
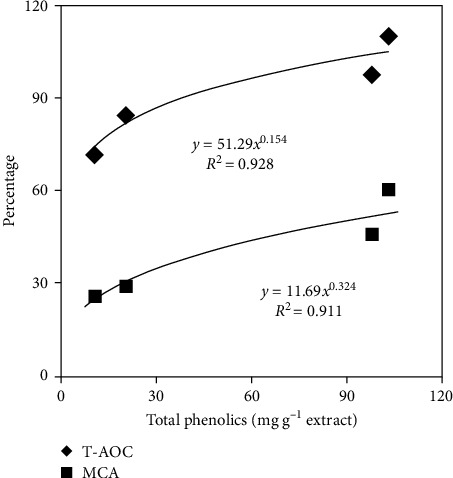
The correlations of total phenolic content with the total antioxidant capacity (T-AOC) and metal chelating ability (MCA) in the extracts of *S. dulcis*. Values are the means of three replicates.

The phenolic content was higher in acetone extract (AE) than others (*P* < 0.05) (Table 2). After receiving AE treatment, the total antioxidant capacity (T-AOC) was considerably increased (*P* < 0.05), followed by EAE, AQE, and PEE. Comparable trend was seen in metal chelating ability (MCA). Power regression of phenolic content in extract of *S. dulcis* on T-AOC and MCA was presented in Figure 2. The T-AOC and MCA showed a positive correlation with the phenolic concentration of SDE.

### 3.2. SDE Depressed Lipid oxidation *In Vitro*

After receiving AE treatment, the PO level was considerably reduced, followed by EAE, AQE, and PEE (*P* < 0.05) (Table 3). Comparable patterns were seen in CD and MDA levels when treated with different extract of *S. dulcis*.

After receiving AE treatment, the PO level was considerably reduced, followed by EAE, AQE, and PEE (*P* < 0.05) (Table 4). Comparable patterns were seen in CD and MDA levels when treated with different extract of *S. dulcis*.

**Figure 3:**
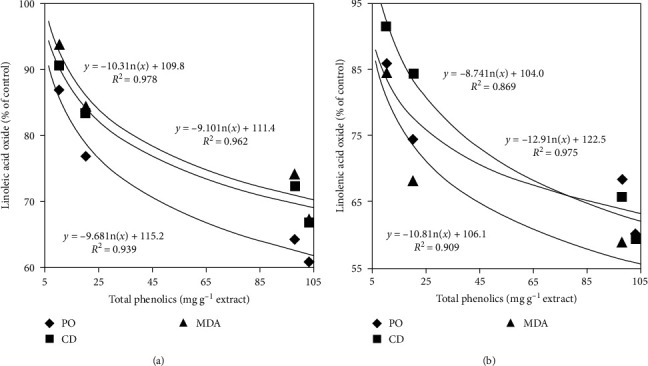
The correlations of total phenolic content in the extracts of *S. dulcis* with the levels of peroxide (PO), conjugated diene (CD), and malonaldehyde (MDA) in linoleic acid emulsion (a) and linolenic acid emulsion (b). The data represent the means of three replicates.

The PO, CD, and MDA levels showed a negative correlation with the phenolic concentration of SDE (Figure 3).

**Figure 4:**
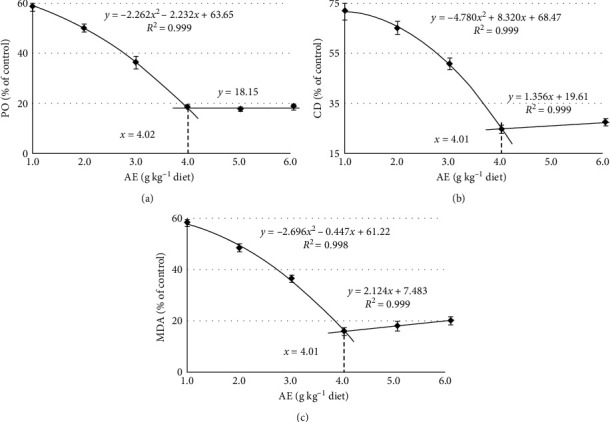
Broken-line analysis of peroxide (PO) (a), conjugated diene (CD) (b), and malondialdehyde (MDA) (c) levels in fish feeds treated with graded levels of acetone extract (AE) of *S. dulcis* for 90 days. Values are the means ± SD of three replicates.

The levels of PO, CD, and MDA were significantly decreased when AE concentration increased up to 4.0 g kg^−1^ (*P* < 0.05), stabilized as dietary AE content increased further (*P* > 0.05) (Figure 4). Broken-line analysis revealed that the optimal AE supplements were 4.02, 4.01, and 4.01 g kg^−1^ determined from PO, CD, and MDA in fish feed, respectively.

### 3.3. Dietary AE Inclusion Enhanced Fish Growth

**Figure 5:**
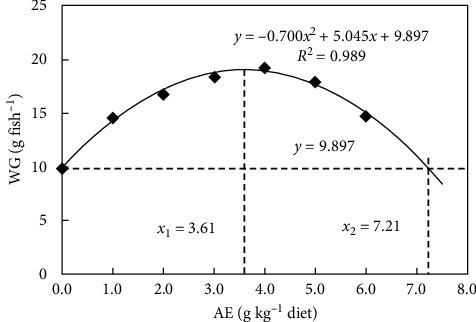
Polynomial regression analysis of weight gain (WG) for Jian carp fed diets containing graded levels of acetone extract (AE) of *S. dulcis* for 60 days. Values are mean ± SD of four replicates.

In Table 5, survival rate was not affected among treatments (*P*  > 0.05). WG and FBW were significantly enhanced when AE concentration increased up to 3.0 g kg^−1^ (*P*  < 0.05), stabilized when AE concentration increased up to 5.0 g kg^−1^ (*P*  > 0.05), and decreased as dietary AE content increased further (*P*  < 0.05). Comparable patterns were seen in SGR and FI. FE was lower in the control group than the AE supplementation groups. Based on polynomial regression analysis, optimal dietary AE supplement was 3.61 g kg^−1^ diet determined from weight gain (Figure 5).

In Table 6, trypsin activities in the hepatopancreas enhanced when AE concentration increased up to 3.0 g kg^−1^ (*P*  < 0.05), stabilized as dietary AE content increased further (*P*  > 0.05). Comparable trends were seen in activities of GOT, CAT, and GPT. Lipase activities in the hepatopancreas enhanced when AE concentration increased up to 5.0 g kg^−1^ (*P*  < 0.05), stabilized as dietary AE content increased further (*P*  > 0.05). Comparable pattern was seen in GPx activity. MDA concentration in the hepatopancreas decreased when AE concentration increased up to 4.0 g kg^−1^ (*P*  < 0.05), stabilized as dietary AE content increased further (*P*  > 0.05).

In Table 7, trypsin activities in the intestine enhanced when AE concentration increased up to 3.0 g kg^−1^ (*P*  < 0.05), stabilized as dietary AE content increased further (*P*  > 0.05). Comparable patterns were seen in activities of lipase, GPT, and amylase. Activities of AKP in the intestine enhanced when AE concentration increased up to 4.0 g kg^−1^ (*P*  < 0.05), stabilized as dietary AE content increased further (*P*  > 0.05). Comparable trends were seen in activities of GOT and CAT. MDA content in the intestine decreased when AE concentration increased up to 4.0 g kg^−1^ (*P*  < 0.05), stabilized as dietary AE content increased further (*P*  > 0.05). GPx activities in the intestine enhanced when AE concentration increased up to 2.0 g kg^−1^ (*P*  < 0.05), stabilized as dietary AE content increased further (*P*  > 0.05).

### 3.4. Effects of Dietary AE on Erythrocytes and Plasma Parameters, the Antioxidant Status in Fish Erythrocytes, Gills and Muscle, and Hypoxia Tolerance

As shown in Table 8, Hct was not influenced among treatments (*P*  > 0.05). RBC enhanced when AE concentration increased up to 3.0 g kg^−1^ (*P*  < 0.05), stabilized as dietary AE content increased further (*P*  > 0.05). Comparable trend was seen in MCH. HbC and MCHC were higher in fish feed diet with 3.0 g kg^−1^ AE. MCV in control group was higher.

As shown in Table 9, the levels of O_2_^•−^ significantly decreased when AE concentration increased up to 5.0 g kg^−1^ (*P*  < 0.05), stabilized as AE concentration increased further (*P*  > 0.05). Comparable trends were seen in H_2_O_2_ and MDA. The levels of Met-Hb significantly decreased with the increase in AE concentration (*P*  < 0.05). GOT activities elevated when AE concentration increased up to 2.0 g kg^−1^ (*P*  < 0.05), stabilized as dietary AE content increased further (*P*  > 0.05). Comparable trends were seen in LDH, CAT, and GSH. The activity of GPT was lower, and PAC was higher in the control group, respectively. The activities of SOD enhanced when AE concentration increased up to 3.0 g kg^−1^ (*P*  < 0.05), stabilized when AE concentration increased up to 5.0 g kg^−1^ (*P*  > 0.05), and decreased as AE concentration increased further (*P*  < 0.05).

As shown in Table 10, ASA capacity in fish gills was enhanced when AE concentration increased up to 2.0 g kg^−1^ (*P*  < 0.05), stabilized as dietary AE content increased further (*P*  > 0.05). Comparable trends were seen in AHR, CAT, GR, GST, and GSH. Th levels of MDA significantly decreased when AE concentration increased up to 4.0 g kg^−1^ (*P*  < 0.05), stabilized as AE concentration increased further (*P*  > 0.05). Comparable pattern was seen in PC.

In Table 11, GOT activity in fish muscle was enhanced when AE concentration increased up to 2.0 g kg^−1^ (*P*  < 0.05), stabilized when AE concentration increased up to 5.0 g kg^−1^ (*P*  > 0.05), and then decreased as dietary AE content increased further (*P*  < 0.05). Comparable trend was seen in GSH. GPT activity in fish muscle was enhanced when AE concentration increased up to 2.0 g kg^−1^ (*P*  < 0.05), stabilized as dietary AE content increased further (*P*  > 0.05). Comparable trends were seen in LDH, SOD, CAT, and GR. The levels of MDA decreased when AE concentration increased up to 4.0 g kg^−1^ (*P*  < 0.05), stabilized as dietary AE content increased further (*P*  > 0.05).

**Figure 6:**
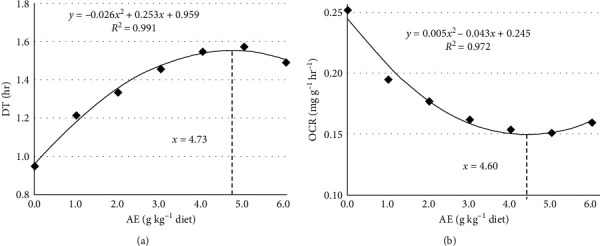
Polynomial regression analysis of durative time (DT) (a) and oxygen consumption rate (OCR) (b) for Jian carp fed diets containing graded levels of acetone extract (AE) of *S. dulcis* for 60 days, followed by treatment with hypoxia. The data represent the means ± SD of three replicates, with five fish in each replicate.

As shown in Table 12, durative time (DT) was enhanced when AE concentration increased up to 3.0 g kg^−1^ (*P*  < 0.05), stabilized as dietary AE content increased further (*P*  > 0.05). Oxygen consumption rate (OCR) was decreased when AE concentration increased up to 3.0 g kg^−1^ (*P*  < 0.05), stabilized as dietary AE content increased further (*P*  > 0.05). Based on polynomial regression analysis, optimal AE supplements were 4.73 and 4.60 g kg^−1^ diet determined from DT and OCR for hypoxia tolerance in Jian carp (Figure 6), respectively.

## 4. Discussion

### 4.1. Relationship between Phenolic Content in SDE and Antioxidant Capacity

*S. dulcis* is a perennial medicinal herb that contains phenolics, flavonoids, tannins, and diterpenoids [23]. According to our most recent research, under stress, fish digestive organ oxidant–antioxidant state was modulated by dietary SDE [16]. The total antioxidant capacity of hydrophilic or lipophilic compounds is represented by the T-AOC, an indication of free radical scavenging action [36, 37]. Based on the ABTS test, the SDE in this research showed T-AOC comparable to trolox. Our study also found that the total phenolic content, T-AOC, and MCA were higher in AE, which indicates that the antioxidant capacity of AE was positively correlated with their total phenolic contents. Similarly, an *in vitro* study showed that phenolic compounds were related to the free radical scavenging potential of SDE [38]. The antioxidant capacity of the phenolic fraction in SDE may be due to the inhibition of xanthine oxidase and lipoxygenase activities, which play an important role in radical generation [39]. However, the mechanism by which SDE regulates antioxidant capacity requires additional investigation.

### 4.2. SDE Inhibited Lipid Oxidation *In Vitro*

In the early stage of the lipid peroxidation process, peroxides and hydroperoxides are the main oxidation products [2]. The conjugated dienes structure of hydroperoxides is generated by resonance following the attack of free radicals on the hydrogen atoms of methylene groups that separate double bonds in peroxidized unsaturated fatty acids [3]. Therefore, PO and CD serve as sensitive markers of primary oxidation [40]. In the secondary stage, peroxides and hydroperoxides react with oxygen or redox metals, finally producing MDA [41]. In this study, AE of SDE reduced PO, CD, and MDA levels in fish feed. Similarly, our recent investigation found that the MDA content was alleviated by dietary SDE supplementation in crucian carp muscle under stress [16]. Our results suggest that adding AE to fish diet may inhibit its lipid oxidation. Therefore, it can be used as a natural antioxidant for fish feed applications. Broken-line analysis revealed that the optimal AE supplements were 4.02, 4.01, and 4.01 g kg^−1^ determined from PO, CD, and MDA in fish feed, respectively.

Lipid oxidation is closely related to the presence of unsaturated fatty acids in fish feed. Linolenic acid and linoleic acid are the essential *ω*-3 and *ω*-6 unsaturated fatty acids in freshwater fishes [42]. In the current investigation, SDE reduced the amounts of PO, CD, and MDA in linoleic and linolenic acid, demonstrating that it could depress lipid oxidation in unsaturated fatty acids by inhibiting the initial stage and interrupting the secondary stage of lipid oxidation. In the tested extracts, AE of SDE was the most effective extract for decreasing lipid oxidation in the linoleic and linolenic acids. This result is consistent with our previous finding that dietary SDE supplementation inhibited the lipid oxidation induced by trichlorfon in fish muscle [16]. In mice, the dietary inclusion of SDE reduced the formation of MDA [26]. The lipid oxidation of unsaturated fatty acids is majorly influenced by ROS [9]. Previous investigations conducted in our lab have found that SDE reduced fish ROS generation, as seen by the decreased hydrogen peroxide (H_2_O_2_) level [16]. The present discovery aligns with the findings described above. These findings showed that by increasing free radical scavenging capability, SDE may protect unsaturated fatty acids from lipid oxidation. The chemical components of SDE may be linked to their beneficial effects on the lipid oxidation in unsaturated fatty acids. The phenolic compounds in *S. dulcis* may significantly contribute to its antilipid peroxidation effect [39]. In the present study, strong relationships were observed between the phenolic concentration in SDE and PO, CD, and MDA levels in unsaturated fatty acids. Furthermore, the high phenolic content in SDE was positively correlated to their T-AOC values, suggesting that phenolic substances in SDE boosted the scavenging activity of free radical. Our investigations suggested that the antioxidant capacity of SDE in aquatic feed may be linked to their phenolic substances.

### 4.3. Dietary AE Inclusion Enhanced Fish Growth

In the present study, the dietary AE supplementation increased FI, WG, and SGR in Jian carp, indicating an improvement in its growth performance. Based on polynomial regression analysis, optimal AE supplement was 3.61 g kg^−1^ diet determined from weight gain. These findings were consistent with our recent study on crucian carp, which found that dietary supplementation of SDE enhanced fish growth performance under stress [16]. Furthermore, the extract of *S. dulcis* can attenuate the reduction in body weight in alloxan-induced stressed rats [24]. Nevertheless, few attentions have focused on the growth-promoting impact of AE in fish.

Fish growth is tightly tied to the process of food digestion, which has been linked to the activity of digestive enzymes [32]. Fish exocrine pancreas produces and releases digestive enzymes (trypsin, chymotrypsin, carbohydrase, and lipase) into the gut [43, 44]. Our study found that AE supplementation resulted in an increase in trypsin, amylase, and lipase in fish's hepatopancreas and intestine, suggesting that it boosted the digestion capacity. In the meantime, fish's growth performance is highly tied to their absorptive ability, which is associated with the activity of brush border membrane enzymes [16]. A crucial enzyme in the absorption of dietary components is AKP, a general indicator of fish absorption ability [45]. Dietary AE supplementation in this study resulted in an increase in AKP activities, suggesting that it enhanced fish's absorption capacity. This finding was consistent our recent observation that SDE treatment raised fish's digestion capacity, as evidenced by the increased activities of amylase and lipase under stress [16]. In MLD-STZ-induced hyperglycemia mice, the dietary inclusion of SDE restored pancreatic histopathological abnormalities [25].

The antioxidant state of aquatic species has a major impact on the functionality and structure of their organs and tissues [46]. The integrity of their intestinal architecture is based on the fish's digestion and absorption [32]. It has been demonstrated that ROS, which are generated during aerobic metabolism or exposure to external chemicals, harm various biomolecules, including proteins and lipids [47]. MDA serves as a sensitive indicator of lipid peroxidation because it is the byproduct of lipid peroxidation [46]. Dietary AE supplementation in this study resulted in a reduction in MDA content in the intestine and hepatopancreas, suggesting that it depressed lipid peroxidation in the digestive and absorptive organs of fish.

The enzymatic antioxidant defense systems in fish have evolved to alleviate the harm caused by oxidative stress because they can scavenge ROS [48]. SOD is the first enzyme in this system to react to O_2_^•−^ and catalyze it to H_2_O_2_ and the dioxygen molecules [46]. Subsequently, H_2_O_2_ is catalyzed by CAT and GPx [49]. In this study, dietary AE supplementation increased activities of CAT and GPx in the hepatopancreas and intestine, indicates that dietary AE improved fish antioxidant capacity. Our results aligned with the report that dietary inclusion of SDE alleviated *λ*-carrageenan-induced paw edema, indicated by the increase of GPx activity in the mice liver [26]. The phenolic compounds in AE may be responsible for its beneficial effects. In LPS-induced mice, the antioxidant enzymes (SOD, GPx, and CAT) activities in the liver were accelerated by the supplementation of curcumin, a phenolic additive [50]. Thus, the results of this investigation showed AE improved fish enzymatic antioxidant capacity, thereby enhancing the radical scavenging ability of digestive and absorptive organs. However, the mechanism underlying the regulation of fish antioxidant capacity by AE requires additional investigation.

### 4.4. Dietary AE Supplementation Improved Fish Hypoxia Tolerance

Fish erythrocytes play a crucial role in supplying oxygen to all tissues and organs by carrying hemoglobin (Hb) [19]. In this study, dietary AE supplementation increased RBC and HbC indicates that dietary AE improved oxygen transport capacity in fish. Compared with mature fish red blood cells, young cells have smaller volume and lower Hb content [51]. Our study found that dietary AE supplementation reduced MCV and MCH, suggesting that dietary AE may improve oxygen transport capacity by increasing the young cell. However, there is few information about the relationship between SDE and fish hematological parameters.

In addition, erythrocytes are sensitive to ROS not only due to the elevated O_2_ tension and heme Fe content but also the high content of PUFA in the membrane [52, 53]. Normally, Hb, an oxygen carrier protein, can be inhibited by ROS through the oxidation of Hb to Met-Hb [53]. Our study found that dietary AE supplementation reduced the content of Met-Hb as well as MDA, superoxide radicals (O_2_^•−^), and H_2_O_2_ in fish erythrocytes, which indicates that dietary AE reduced the production of ROS and depressed lipid peroxidation fish erythrocytes. Our previous study *in vitro* showed that glutamine alleviated oxidative stress by reducing Met-Hb formation in fish erythrocytes [35]. Similar observations were reported in our laboratory that SDE decreased H_2_O_2_ levels in high stocking density-induced stressed fish [16]. In addition, dietary AE supplementation raised GSH content and the activities of SOD and CAT in fish erythrocytes, indicating that dietary AE regulated antioxidant defense systems, which in turn decreased lipid peroxidation and ROS generation. However, the mechanism by which AE regulated the antioxidant status in fish erythrocytes needs to be further explored.

Fish gills, as important respiratory and osmoregulatory organs, have high erythrocyte and Hb levels and are more vulnerable to ROS than other organs due to their exposure to environmental stress [54]. In this study, dietary AE supplementation decreased MDA levels, sensitive maker of lipid peroxidation, suggesting that AE could depress lipid peroxidation in fish gills and muscle. In fish, ROS not only induced lipid peroxidation but also contributed to protein oxidation [47]. In this study, dietary AE supplementation decreased PC levels, a sensitive marker of protein oxidation, which indicates that AE could also depress protein oxidation in fish gills and muscle. Hydroxyl radicals (^•^OH), H_2_O_2_, and superoxide radicals (O_2_^•−^) are important ROS [4]. Dietary AE supplementation enhanced AHR and ASA capacity (indicator of ^•^OH and O_2_^•−^-scavenging ability, respectively), which suggested AE supplementation could reduce ROS production in fish gills, therefore depressing lipid peroxidation and protein oxidation. Similar *in vitro* studies have shown that SDE can mitigate the generation of hydroxyl radicals [55].

In order to reverse the damage caused by ROS, ROS scavenging enzymes and nonenzymatic antioxidants (like GSH) were developed in fish [20]. Our study found that dietary AE supplementation boosted CAT activities in fish gills and muscle, which indicates that AE could alleviate the damage of H_2_O_2_ to fish gills and muscle. The maintenance of antioxidant status of glutathione is crucial for preventing cell and body damage caused by free radical [56]. The glutathione antioxidant system, a protective mechanism within cells against oxidative damage, is composed of GST and GR, together with the low-molecular-weight compound known as GSH [57]. In the antioxidant enzyme systems, GST is a class of enzymes with multiple functions which catalyze the electrophilic conjugation metabolites to GSH [58]. GR catalyzes the reduction of glutathione to GSH with NADPH as an electron source [59]. Our study found that dietary AE supplementation enhanced GR activity and GSH content in fish gills and muscle and enhanced GST activity in fish gills, which indicates that dietary AE enhances antioxidant capacity both enzymatically and nonenzymatically. These results were consistent with our previous observation that dietary inclusion of SDE restored the activities of SOD, CAT, and GPx in fish muscle under trichlorfon exposure [16]. Our current study found that AE improved antioxidant capacity both enzymatically and nonenzymatically, enhancing the radical scavenging ability in fish gills and muscles. Nevertheless, the molecular mechanism via which AE regulates fish antioxidant capability requires additional investigation.

In fish farming, the DO is a key factor due to their aerobic metabolism [14]. In practice, oxygen deficiency (also called hypoxia) is a normal event resulting from daily fluctuations of oxygen content in aquaculture water [15]. Hence, fish are frequently experienced conditions of hypoxia/reoxygenation. In this study, dietary AE supplementation increased durative time (DT) under hypoxia condition, which indicates that dietary AE improved the tolerance to low oxygen level. To cope with low oxygen conditions, fish typically decreases their metabolic rate [60]. In this study, dietary AE supplementation decreased oxygen consumption rate (OCR) under hypoxia condition, which suggests that dietary AE may depress oxygen consumption so as to improving the tolerance ability. Fish always uses protein for energy supply. In teleost fish, GOT and GPT are two crucial protein metabolic enzymes, and the final byproduct of protein metabolism is ammonia [32]. In our study, dietary AE supplementation increased GOT and GPT activities in fish hepatopancreas, intestine, and erythrocyte while decreasing PAC in muscles. This indicates that dietary AE regulates protein metabolism in different fish organs and cells. To reduce the energy demands, animals employ anaerobic glycolysis as the ATP generation pathway [61]. In the anaerobic metabolism, LDH catalyzes pyruvate to lactate and produces energy [62]. Our study found that dietary AE supplementation boosted LDH activity in fish erythrocyte and muscle, suggesting that dietary AE regulated energy metabolism. Therefore, our results suggest that dietary AE improves the hypoxia tolerance ability, contributing considerably to reducing oxygen consumption and regulation of protein and energy metabolism. More research is needed to understand how AE regulated the hypoxia tolerance ability in fish. Based on polynomial regression analysis, optimal dietary AE supplements were 4.73 and 4.60 g kg^−1^ diet determined from DT and OCR for hypoxia tolerance in Jian carp, respectively.

## 5. Conclusion

Therefore, we conclude that SDE's antioxidant ability was highly correlated with its total phenolic concentration. Meanwhile, SDE could depress lipid peroxidation in unsaturated fatty acids and fish feed. Broken-line analysis revealed that the optimal AE supplements were 4.02, 4.01, and 4.01 g kg^−1^ determined from PO, CD, and MDA in fish feed, respectively. In addition, dietary AE supplementation improved fish growth performance. Based on polynomial regression analysis, optimal dietary AE supplement was 3.61 g kg^−1^ diet determined from weight gain. Dietary AE supplementation enhanced fish digestive and absorptive ability as well as antioxidant status in fish digestive organs, which may explain the beneficial effects of AE on fish growth performance. In addition, dietary AE supplementation enhanced antioxidant defense systems by increasing the activities of antioxidant enzymes and the levels of nonenzymatic antioxidants in fish erythrocyte, gills, and muscle. Meanwhile, dietary AE supplementation improved the hypoxia tolerance ability. Based on polynomial regression analysis, optimal dietary AE supplements were 4.73 and 4.60 g kg^−1^ diet determined from DT and OCR for hypoxia tolerance in fish, respectively. Our present study indicates that the phenolic compounds of AE may play an important function in antioxidant capacity. Further *in vitro* studies can focus in understanding the molecular mechanism by which phenolic compounds in SDE improve the antioxidant capacity, thereby alleviating the detrimental effects of hypoxia and lipid oxidation.

## Figures and Tables

**Table 1:** The composition analyses of acetone extract of *S. dulcis* by gas chromatograph–mass spectrometer (GC-MS).

Retention time (min)	Compound name	Molecular weight (amu)	Molecular formula	Matching degree (%)
7.290	Bicyclo (3.1.1)heptane, 2,6,6-trimethyl-	138	C_10_H_18_	89
7.447	6-Methoxy-2-benzoxazolinone	165	C_8_H_7_NO_3_	83
8.485	Hexadecanoic acid, ethyl ester	284	C_18_H_36_O_2_	80

9.494	n-Hexadecanoic acid	256	C_16_H_32_O_2_	99
	Tridecanoic acid	214	C_13_H_26_O_2_	93
	Octadecanoic acid	284	C_18_H_36_O_2_	87
	Tetradecanoic acid	228	C_14_H_28_O_2_	81

This experiment was repeated three times with similar results achieved.

**Table 2:** The total phenolic content, total antioxidant capacity (T-AOC), and metal chelating ability (MCA) of petroleum ether extract (PEE), ethyl acetate extract (EAE), acetone extract (AE), and aqueous extract (AQE) of *S. dulcis*.

Extracts	Phenolics (mg g^−1^ dry extracts)	T-AOC (% of 1 mM trolox)	MCA (% of control)
PEE	10.3 ± 0.83^a^	72.06 ± 3.35^a^	26.29 ± 2^a^
EAE	97.97 ± 7.39^c^	98.19 ± 2.07^c^	46.01 ± 2.26^b^
AE	103.31 ± 4.89^c^	110.38 ± 3.9^d^	60.56 ± 3.92^c^
AQE	20.27 ± 1.68^b^	84.29 ± 2.37^b^	28.99 ± 2.03^a^

Within the same column, values with different superscripts are significantly different (*P*  < 0.05), and the data represent the means ± SD of three replicates.

**Table 3:** The peroxide (PO), conjugated diene (CD), and malonaldehyde (MDA) levels in a linoleic acid emulsion treated with 1.0 mg mL^−1^ of petroleum ether extract (PEE), ethyl acetate extract (EAE), acetone extract (AE), and aqueous extract (AQE) of *S. dulcis*.

Treatment	PO (% of control)	CD (% of control)	MDA (% of control)
PEE	87.02 ± 1.66^c^	90.7 ± 3.99^d^	93.79 ± 2.42^d^
EAE	64.36 ± 0.99^a^	72.38 ± 3.22^b^	74.35 ± 2.32^b^
AE	60.84 ± 4.07^a^	66.93 ± 2.79^a^	67.48 ± 2.52^a^
AQE	77.01 ± 1.49^b^	83.4 ± 0.46^c^	84.48 ± 1.58^c^

Values within the same column with different superscripts are significantly different (*P*  < 0.05). The data represent the means ± SD of three replicates.

**Table 4:** The peroxide (PO), conjugated diene (CD), and malonaldehyde (MDA) levels in a linolenic acid emulsion treated with 1.0 mg mL^−1^ of petroleum ether extract (PEE), ethyl acetate extract (EAE), acetone extract (AE), and aqueous extract (AQE) of *S. dulcis*.

Treatment	PO (% of control)	CD (% of control)	MDA (% of control)
PEE	85.84 ± 1.15^d^	91.46 ± 1.71^d^	84.41 ± 1.48^c^
EAE	68.3 ± 0.86^b^	65.63 ± 2.64^b^	58.76 ± 1.38^a^
AE	60.13 ± 2.14^a^	59.4 ± 0.72^a^	54.84 ± 1.57^a^
AQE	74.4 ± 0.82^c^	84.43 ± 0.92^c^	68.22 ± 1.89^b^

Values within the same column with different superscripts are significantly different (*P*  < 0.05). The data represent the means ± SD of three replicates.

**Table 5:** Initial body weight (IBW), final body weight (FBW), weight gain (WG), specific growth rate (SGR), feed intake (FI), feed efficiency (FE), and survival rate (SR) of Jian carp fed diets containing graded levels of acetone extract (AE) of *S. dulcis* for 60 days.

AE (g kg^−1^ diet)	IBW (g fish^−1^)	FBW (g fish^−1^)	WG (g fish^−1^)	SGR (% d^−1^)	FI (g fish^−1^)	FE (%)	SR (%)
0.0	4.33 ± 0.16^a^	14.19 ± 0.89^a^	9.83 ± 0.69^a^	1.98 ± 0.12^a^	19.64 ± 0.84^a^	50.08 ± 3.52^a^	100.00 ± 0.00^a^
1.0	4.31 ± 0.09^a^	18.97 ± 1.31^b^	14.64 ± 1.12^b^	2.47 ± 0.15^b^	24.54 ± 1.47^b^	59.60 ± 2.79^b^	100.00 ± 0.00^a^
2.0	4.30 ± 0.17^a^	21.17 ± 1.29^c^	16.83 ± 1.29^c^	2.66 ± 0.14^bc^	27.79 ± 1.87^c^	60.54 ± 2.77^b^	100.00 ± 0.00^a^
3.0	4.29 ± 0.15^a^	22.73 ± 1.41^cd^	18.44 ± 1.43^cd^	2.78 ± 0.12^c^	28.46 ± 2.24^c^	64.82 ± 2.89^b^	100.00 ± 0.00^a^
4.0	4.31 ± 0.11^a^	23.58 ± 1.54^d^	19.27 ± 1.56^d^	2.83 ± 0.12^c^	29.01 ± 2.33^c^	66.83 ± 5.16^b^	100.00 ± 0.00^a^
5.0	4.32 ± 0.14^a^	22.27 ± 1.55^cd^	17.95 ± 1.35^cd^	2.73 ± 0.16^c^	27.72 ± 1.83^c^	65.04 ± 5.22^b^	100.00 ± 0.00^a^
6.0	4.29 ± 0.16^a^	19.08 ± 1.32^b^	14.79 ± 1.17^b^	2.48 ± 0.06^b^	24.52 ± 1.60^b^	60.30 ± 3.01^b^	100.00 ± 0.00^a^

Values are mean ± SD of four replicates. Values in the same column with the different superscripts are significantly different (*P*  < 0.05).

**Table 6:** The activities of trypsin, lipase, glutamate–oxaloacetate transaminase (GOT), glutamate–pyruvate transaminase (GPT), catalase (CAT), and glutathione peroxidase (GPx) and the level of malondialdehyde (MDA) in the hepatopancreas of Jian carp fed diets containing different levels of acetone extract (AE) of *S. dulcis* for 60 days.

AE (g kg^−1^ diet)	Trypsin (U mg^−1^ protein)	Lipase (U mg^−1^ protein)	GOT (U g^−1^ protein)	GPT (U g^−1^ protein)	MDA (nmol mg^−1^ protein)	CAT (U mg^−1^ protein)	GPx (U mg^−1^ protein)
0.0	955.2 ± 55.16^a^	31.33 ± 1.55^a^	25.14 ± 1.31^a^	17.77 ± 0.97^a^	9.57 ± 0.59^c^	23.94 ± 1.78^a^	238.27 ± 15.56^a^
1.0	1086.57 ± 44.92^b^	32.77 ± 1.53^a^	26.01 ± 1.32^a^	18.21 ± 0.73^a^	9.2 ± 0.41^c^	26.42 ± 1.67^ab^	251.32 ± 11.71^ab^
2.0	1164.52 ± 76.11^bc^	37.82 ± 2.98^b^	26.41 ± 1.74^a^	21.23 ± 1.32^b^	8.86 ± 0.34^c^	28.92 ± 1.59^bc^	262.25 ± 19.82^abc^
3.0	1258.05 ± 69.53^cd^	40.07 ± 3.02^b^	32.07 ± 2.09^b^	23.47 ± 1.36^bc^	7.92 ± 0.48^b^	31.46 ± 1.23^cd^	275.44 ± 16.95^bc^
4.0	1309.26 ± 76.23^d^	42.18 ± 2.98^bc^	34.06 ± 2.68^b^	25.16 ± 1.9^c^	7.26 ± 0.43^ab^	32.77 ± 2.84^d^	276.98 ± 9.07^bc^
5.0	1313.54 ± 60.17^d^	50.04 ± 4.1^d^	35.68 ± 1.64^b^	25.44 ± 2.2^c^	6.85 ± 0.3^a^	31.08 ± 2.3^cd^	308.03 ± 16.53^d^
6.0	1373.81 ± 99.31^d^	45.76 ± 2.99^cd^	32.79 ± 2.29^b^	23.92 ± 2.11^bc^	6.99 ± 0.44^a^	30.11 ± 1.87^cd^	284.39 ± 12.67^cd^

Values are mean ± SD of three replicates, with five fish in each replicate. Values with the different superscripts in the same column are significantly different (*P*  < 0.05).

**Table 7:** The activities of trypsin, lipase, amylase, alkaline phosphatase (AKP), glutamate–oxaloacetate transaminase (GOT), glutamate–pyruvate transaminase (GPT), catalase (CAT), and glutathione peroxidase (GPx) and the content of malondialdehyde (MDA) in the intestine of Jian carp fed diets containing different levels of acetone extract (AE) of *S. dulcis* for 60 days.

AE (g kg^−1^ diet)	Trypsin (U mg^−1^ protein)	Lipase (U g^−1^ protein)	Amylase (U mg^−1^ protein)	AKP (U g^−1^ protein)	GOT (U g^−1^ protein)
0.0	1,134.24 ± 65.05^a^	32.39 ± 2.16^a^	1.19 ± 0.09^a^	178.83 ± 10.22^a^	12.46 ± 0.71^a^
1.0	1,341.3 ± 102.44^b^	34.61 ± 2.14^a^	1.21 ± 0.08^a^	179.45 ± 10.5^a^	13.81 ± 0.93^a^
2.0	1,333.79 ± 119.49^b^	35.6 ± 2.2^ab^	1.2 ± 0.08^a^	209.12 ± 15.29^b^	16.13 ± 0.78^b^
3.0	1,441.66 ± 107.57^bc^	39.02 ± 2.18^bc^	1.32 ± 0.1^ab^	256.93 ± 15.66^c^	15.97 ± 0.77^b^
4.0	1,578.13 ± 136.88^c^	41.2 ± 2.1^c^	1.38 ± 0.11^ab^	289.8 ± 16.8^d^	17.23 ± 1.36^bc^
5.0	1,539.85 ± 106.92^bc^	41.28 ± 2.23^c^	1.47 ± 0.09^b^	313.45 ± 12.97^d^	17.88 ± 0.75^c^
6.0	1,406.52 ± 106.07^bc^	39.71 ± 2.15^c^	1.3 ± 0.08^ab^	307.01 ± 10.18^d^	17.03 ± 0.63^bc^

AE (g kg^−1^ diet)	GPT (U g^−1^ protein)	MDA (nmol mg^−1^ protein)	CAT (U mg^−1^ protein)	GPx (U mg^−1^ protein)	

0.0	8.06 ± 0.43^a^	11.04 ± 0.66^c^	11.55 ± 0.89^a^	276.1 ± 16.73^a^	
1.0	9.23 ± 0.74^a^	10.3 ± 0.51^bc^	12.06 ± 0.78^a^	298.8 ± 21.96^ab^	
2.0	11.81 ± 0.78^b^	10.14 ± 0.71^bc^	14.19 ± 0.77^b^	324.45 ± 14.79^bc^	
3.0	13.5 ± 0.9^c^	9.68 ± 0.36^b^	15.15 ± 0.76^b^	323.98 ± 12.91^bc^	
4.0	15.03 ± 0.77^c^	8.48 ± 0.37^a^	17.64 ± 1.16^c^	341.8 ± 14.1^c^	
5.0	14.65 ± 0.93^c^	8.24 ± 0.61^a^	18.47 ± 1.13^c^	352.28 ± 20.01^c^	
6.0	14.35 ± 1.19^c^	8.36 ± 0.56^a^	17.52 ± 0.65^c^	344.44 ± 20.97^c^	

Values are mean ± SD of three replicates, with five fish in each replicate. Values with different superscripts in the same column are significantly different (*P*  < 0.05).

**Table 8:** The hematocrit (Hct), red blood corpuscle count (RBC), hemoglobin concentration (HbC), mean corpuscular volume (MCV), mean corpuscular hemoglobin content (MCH), and mean corpuscular hemoglobin concentration (MCHC) in blood of Jian carp fed diets containing different levels of acetone extract (AE) of *S. dulcis* for 60 days.

AE (g kg^−1^ diet)	Hct (%)	RBC (10^12^ L^−1^)	HbC (g L^−1^)	MCV (fL cell^−1^)	MCH (pg cell^−1^)	MCHC (g L^−1^)
0.0	51.23 ± 2.9^a^	2.46 ± 0.03^a^	94.34 ± 3.11^a^	207.97 ± 10.36^b^	38.29 ± 0.78^c^	184.34 ± 6.48^a^
1.0	52.3 ± 3.23^a^	2.64 ± 0.13^ab^	100.84 ± 5.08^ab^	198.19 ± 7.18^ab^	38.21 ± 0.48^c^	193.04 ± 9.28^abc^
2.0	53.33 ± 3.55^a^	2.79 ± 0.1^bc^	107.1 ± 3.66^bcd^	191.48 ± 16.54^ab^	38.43 ± 2^c^	201.13 ± 7.33^bc^
3.0	56.85 ± 2.4^a^	3.13 ± 0.18^d^	116.52 ± 6^d^	182.24 ± 15.88^a^	37.26 ± 1.53^bc^	205.18 ± 13.24^c^
4.0	56.17 ± 2.76^a^	3.2 ± 0.16^d^	112.55 ± 7.58^cd^	175.61 ± 6.58^a^	35.16 ± 1.21^ab^	200.27 ± 4.68^bc^
5.0	54.72 ± 3.43^a^	3.04 ± 0.23^cd^	103.95 ± 5.68^abc^	180.12 ± 11.49^a^	34.2 ± 1.14^a^	190.1 ± 6.14^abc^
6.0	53.92 ± 3.25^a^	2.96 ± 0.12^cd^	100.94 ± 3.71^ab^	182.2 ± 10.4^a^	34.13 ± 1.7^a^	187.4 ± 5.2^ab^

Values are means ± SD of three replicates, with five fish in each replicate. Values in the same column with different superscripts are significantly different (*P* < 0.05).

**Table 9:** The levels of superoxide anion (O_2_^·−^), hydrogen peroxide (H_2_O_2_), met-hemoglobin (Met-Hb), malondialdehyde (MDA), and reduced glutathione (GSH) and the activities of glutamate–oxaloacetate transaminase (GOT), glutamate–pyruvate transaminase (GPT), lactate dehydrogenase (LDH), superoxide dismutase (SOD), and catalase (CAT) in erythrocytes of and plasma ammonia content (PAC) of Jian carp fed diets containing different levels of acetone extract (AE) of *S. dulcis* for 60 days.

AE (g kg^−1^ diet)	O_2_^·-^ (U g^−1^ protein)	H_2_O_2_ (mmol g^−1^ protein)	Met-Hb (g L^−1^)	GOT (U g^−1^ protein)	GPT (U g^−1^ protein)	PAC (*μ*mol mL^−1^)
0.0	31.56 ± 1.84^e^	46.21 ± 1.96^d^	2.76 ± 0.14^e^	16.48 ± 1.07^a^	17.17 ± 1.15^a^	317.31 ± 17.65^b^
1.0	30.94 ± 1.19^de^	44.11 ± 2.49^cd^	2.6 ± 0.17^de^	17.06 ± 1.01^ab^	18.08 ± 1.18^ab^	294.59 ± 19.05^ab^
2.0	28.47 ± 1.79^cd^	41.18 ± 1.62^c^	2.45 ± 0.06^cd^	17.72 ± 0.88^abc^	17.96 ± 1.47^ab^	285.99 ± 18.27^ab^
3.0	27.14 ± 1.02^c^	36.8 ± 1^b^	2.4 ± 0.08^cd^	18.87 ± 1.16^bc^	18.94 ± 0.58^ab^	286.33 ± 16.14^ab^
4.0	23.74 ± 1.53^b^	31.44 ± 2.47^a^	2.27 ± 0.16^bc^	19.41 ± 0.96^c^	19.46 ± 0.91^b^	278.42 ± 19.16^a^
5.0	20.72 ± 1.46^a^	32.86 ± 2.73^a^	2.05 ± 0.17^b^	18.66 ± 1.07^bc^	18.94 ± 0.9^ab^	277.73 ± 18.27^a^
6.0	20.37 ± 0.67^a^	33.33 ± 1.63^ab^	1.76 ± 0.04^a^	18.63 ± 0.47^bc^	18.53 ± 1.04^ab^	276.35 ± 20.03^a^

AE (g kg^−1^ diet)	LDH (U g^−1^ protein)	MDA (nmol mg^−1^ protein)	SOD (U mg^−1^ protein)	CAT (U mg^−1^ protein)	GSH (mg g^−1^ protein)	

0.0	82.9 ± 3.26^a^	6.14 ± 0.37^c^	71.07 ± 3.35^a^	5.81 ± 0.45^a^	5.43 ± 0.34^a^	
1.0	85.29 ± 5.69^ab^	5.95 ± 0.19^c^	80.88 ± 2.69^b^	6.29 ± 0.45^a^	5.68 ± 0.34^ab^	
2.0	91.77 ± 3.24^abc^	6.01 ± 0.24^c^	84.16 ± 3.18^bc^	7.85 ± 0.26^b^	5.91 ± 0.22^abc^	
3.0	93.99 ± 6.64^abc^	5.38 ± 0.18^b^	90.94 ± 3.35^cde^	8.79 ± 0.26^d^	6.12 ± 0.34^bc^	
4.0	100.97 ± 8.9^c^	4.43 ± 0.36^a^	95.87 ± 5.3^e^	8.9 ± 0.53^d^	6.43 ± 0.35^cd^	
5.0	97.83 ± 5.75^c^	4.4 ± 0.18^a^	92.48 ± 5.99^de^	9.4 ± 0.53^d^	7.02 ± 0.47^d^	
6.0	94.76 ± 6.57^bc^	4.45 ± 0.06^a^	86.68 ± 3.47^bcd^	8.84 ± 0.26^d^	6.94 ± 0.34^d^	

Values are means ± SD of three replicates, with five fish in each replicate. Values in the same column with different superscripts are significantly different (*P* < 0.05).

**Table 10:** The levels of malondialdehyde (MDA), protein carbonyl (PC), and reduced glutathione (GSH) and the activities of antisuperoxide anion (ASA), antihydroxy radical (AHR), catalase (CAT), glutathione reductase (GR), and glutathione-*S*-transferase (GST) in gills of Jian carp fed diets containing different levels of acetone extract (AE) of *S. dulcis* for 60 days.

AE (g kg^−1^ diet)	ASA (U g^−1^ protein)	AHR (U mg^−1^ protein)	MDA (nmol mg^−1^ protein)	PC (nmol mg^−1^ protein)	CAT (U mg^−1^ protein)	GSH (mg g^−1^ protein)	GR (U g^−1^ protein)	GST (U g^−1^ protein)
0.0	55.27 ± 2.36^a^	22.15 ± 1.1^a^	10.16 ± 0.58^c^	7.87 ± 0.59^c^	6.98 ± 0.24^a^	7.95 ± 0.44^a^	12.05 ± 0.27^a^	38.68 ± 2.5^a^
1.0	57.34 ± 2.97^a^	28.04 ± 1.18^b^	9.45 ± 0.32^bc^	7.28 ± 0.51^c^	7.57 ± 0.32^ab^	8.12 ± 0.44^ab^	12.92 ± 0.56^ab^	40.01 ± 2.57^a^
2.0	60.88 ± 3.6^ab^	27.94 ± 2.36^b^	8.77 ± 0.31^b^	7.13 ± 0.44^c^	7.54 ± 0.23^ab^	8.6 ± 0.44^abc^	13.6 ± 0.41^bc^	39.87 ± 2.15^a^
3.0	60.47 ± 4.81^ab^	32.68 ± 1.95^c^	8.8 ± 0.61^b^	6.3 ± 0.34^b^	7.88 ± 0.5^ab^	8.87 ± 0.45^bcd^	14.3 ± 0.9^cd^	44.79 ± 2.37^b^
4.0	64.69 ± 2.38^b^	36.1 ± 3.18^c^	7.87 ± 0.47^a^	5.32 ± 0.41^a^	8.4 ± 0.32^bc^	9.53 ± 0.45^d^	14.68 ± 0.64^cd^	50.42 ± 2.24^c^
5.0	64.81 ± 3.47^b^	35.96 ± 2.83^c^	7.55 ± 0.57^a^	5.2 ± 0.23^a^	9.25 ± 0.41^c^	9.67 ± 0.45^d^	16.1 ± 0.58^e^	48.24 ± 2.65^bc^
6.0	61.54 ± 4.6^ab^	34.81 ± 3.08^c^	7.23 ± 0.49^a^	5.53 ± 0.29^a^	8.89 ± 0.74^c^	9.23 ± 0.43^cd^	15.29 ± 0.77^de^	46.69 ± 3.71^bc^

Values are mean ± SD of three replicates, with five fish in each replicate. Values with different superscripts in the same column are significantly different (*P* < 0.05).

**Table 11:** The level of malondialdehyde (MDA) and reduced glutathione (GSH) and the activities of glutamate–oxaloacetate transaminase (GOT), glutamate–pyruvate transaminase (GPT), lactate dehydrogenase (LDH), superoxide dismutase (SOD), catalase (CAT), and glutathione reducase (GR) in the muscle of Jian carp fed diets containing different levels of acetone extract (AE) of *S. dulcis* for 60 days.

AE (g kg^−1^ diet)	GOT (U g^−1^ protein)	GPT (U g^−1^ protein)	LDH (U g^−1^ protein)	MDA (nmol mg^−1^ protein)	SOD (U mg^−1^ protein)	CAT (U mg^−1^ protein)	GSH (mg g^−1^ protein)	GR (U g^−1^ protein)
0.0	34.57 ± 2.12^a^	14.94 ± 1^a^	239.38 ± 17.1^a^	7.8 ± 0.46^e^	83.57 ± 5.14^a^	17.3 ± 0.94^a^	5.19 ± 0.24^a^	29.31 ± 1.34^a^
1.0	36.49 ± 2.24^ab^	16.33 ± 0.51^a^	260.21 ± 17.35^ab^	6.67 ± 0.32^d^	90.92 ± 7.36^ab^	18.65 ± 0.95^ab^	6.69 ± 0.25^b^	32.52 ± 1.38^ab^
2.0	40.66 ± 1.75^bcd^	21.84 ± 1.41^b^	284.38 ± 10.05^bc^	5.81 ± 0.21^c^	102.56 ± 5.57^b^	20.37 ± 0.95^b^	7.00 ± 0.25^bc^	35.34 ± 2.47^bc^
3.0	42.09 ± 3.03^cd^	23.4 ± 1.41^b^	297.98 ± 19.85^cd^	5.1 ± 0.26^b^	101.03 ± 8.69^b^	23.91 ± 1.88^c^	8.18 ± 0.49^d^	35.1 ± 2.14^bc^
4.0	44.28 ± 3^cd^	23.72 ± 1.4^b^	294.26 ± 9.8^cd^	4.39 ± 0.22^a^	125.19 ± 5.71^c^	29.52 ± 2.46^d^	8.35 ± 0.42^d^	37.82 ± 2.83^c^
5.0	44.69 ± 2.44^d^	22.6 ± 0.95^b^	313.91 ± 10.26^d^	4.34 ± 0.3^a^	144.58 ± 7.26^d^	28.65 ± 1.69^d^	8.16 ± 0.5^d^	36.74 ± 2.39^c^
6.0	39.68 ± 2.33^bc^	22 ± 1.04^b^	302.88 ± 16.83^cd^	4.11 ± 0.25^a^	137.39 ± 8.51^cd^	27.66 ± 1.84^d^	7.45 ± 0.41^c^	35.02 ± 1.84^bc^

Values are mean ± SD of three replicates, with five fish in each replicate. Values with different superscripts in the same column are significantly different (*P* < 0.05).

**Table 12:** Body weight, water volume, initial dissolved oxygen (IDO), durative time (DT), final dissolved oxygen (FDO), and oxygen consumption rate (OCR) in Jian carp fed diets containing different levels of acetone extract (AE) of *S. dulcis* for 60 days, followed by treatment with hypoxia.

AE (g kg^−1^ diet)	Body weight (g fish^−1^)	Water volume (L bottle^−1^)	IDO (mg L^−1^)	DT (h)	FDO (mg L^−1^)	OCR (mg g^−1^ h^−1^)
0.0	15.85 ± 0.28^a^	4.28 ± 0.08^a^	8.03 ± 0.11^a^	0.95 ± 0.08^a^	0.09 ± 0.01^a^	0.252 ± 0.023^d^
1.0	15.70 ± 0.28^a^	4.24 ± 0.08^a^	7.96 ± 0.11^a^	1.22 ± 0.1^b^	0.10 ± 0.02^a^	0.195 ± 0.018^c^
2.0	15.89 ± 0.29^a^	4.29 ± 0.08^a^	7.95 ± 0.06^a^	1.33 ± 0.06^bc^	0.09 ± 0.01^a^	0.177 ± 0.007^bc^
3.0	16.04 ± 0.28^a^	4.33 ± 0.08^a^	7.96 ± 0.08^a^	1.46 ± 0.07^cd^	0.10 ± 0.01^a^	0.162 ± 0.007^ab^
4.0	16.07 ± 0.34^a^	4.34 ± 0.09^a^	7.99 ± 0.08^a^	1.55 ± 0.06^d^	0.09 ± 0.01^a^	0.153 ± 0.007^a^
5.0	16.15 ± 0.26^a^	4.36 ± 0.07^a^	8.00 ± 0.06^a^	1.57 ± 0.1^d^	0.10 ± 0.01^a^	0.151 ± 0.008^a^
6.0	16.00 ± 0.29^a^	4.32 ± 0.08^a^	8.02 ± 0.06^a^	1.49 ± 0.06^d^	0.09 ± 0.01^a^	0.159 ± 0.006^ab^

Values are means ± SD of three replicates, with nine fish in each replicate. Values in the same column with the different superscripts are significantly different (*P* < 0.05).

## Data Availability

Data supporting the findings of this study are available from the corresponding author upon reasonable request.
